# Intellectual Profiles in KBG-Syndrome: A Wechsler Based Case-Control Study

**DOI:** 10.3389/fnbeh.2017.00248

**Published:** 2017-12-19

**Authors:** Linde C. M. van Dongen, Ellen Wingbermühle, Wouter Oomens, Anja G. Bos-Roubos, Charlotte W. Ockeloen, Tjitske Kleefstra, Jos I. M. Egger

**Affiliations:** ^1^Centre of Excellence for Neuropsychiatry, Vincent van Gogh Institute for Psychiatry, Venray, Netherlands; ^2^Donders Institute for Brain, Cognition and Behaviour, Radboud University Nijmegen, Nijmegen, Netherlands; ^3^Department of Human Genetics, Radboud University Medical Centre, Nijmegen, Netherlands; ^4^Centre of Excellence for Korsakoff and Alcohol-Related Cognitive Disorders, Vincent van Gogh Institute for Psychiatry, Venray, Netherlands

**Keywords:** ANKRD11, neurodevelopmental disorder, KBG syndrome, intelligence, cognition, Wechsler scales, contextual neuropsychology, case-control study

## Abstract

KBG syndrome is a neurodevelopmental disorder (NDD) caused by loss-of-function of the *ANKRD11* gene. The core phenotype comprises developmental delay (DD)/ intellectual disability (ID) and several specific facial dysmorphisms. In addition, both ADHD- and ASD-related symptoms have been mentioned. For the correct understanding of these developmental and behavioral characteristics however, it is of great importance to apply objective measures, which seldom has been done in patients with KBG syndrome. In this study, intelligence profiles of patients with KBG syndrome (*n* = 18) were compared with a control group comprising patients with NDD caused by various other genetic defects (*n* = 17), by means of the Wechsler scales. These scales were also used to measure speed of information processing, working memory, verbal comprehension and perceptual reasoning. No significant differences were found in the global level of intelligence of patients with KBG syndrome as compared to the patient genetic control group. The same was true for Wechsler subtest results. Hence, behavioral problems associated with KBG syndrome cannot directly be related to or explained by a specific intelligence profile. Instead, specific assessment of neurocognitive functions should be performed to clarify the putative behavioral problems as observed in this syndrome.

## Introduction

KBG syndrome, caused by loss-of-function of the Ankyrin repeat domain-containing protein 11 gene (*ANKRD11)*, is a neurodevelopmental disorder (NDD) named after the surname initials (K, B and G) of the first three families with KBG syndrome, and first described by Herrmann et al. (1975). Today, approximately 180 patients have been reported worldwide (Sirmaci et al., 2011; Ockeloen et al., 2015; Walz et al., 2015; Goldenberg et al., 2016; Low et al., 2016). *ANKRD11* appeared one of the most frequently mutated genes in a study of 4293 children with severe developmental disorders. Accordingly, KBG syndrome appears to be much more common than previously recognized (The Deciphering Developmental Disorders Study, 2015).

Although clinical features may vary, the core symptoms of KBG syndrome are developmental delay (DD)/intellectual disability (ID), dental anomalies, triangular facies, brachycephaly, hypertelorism, protruding ears and an upturned nose with full nasal tip. In addition, KBG syndrome is frequently associated with hearing problems, short stature as well as cardiac defects, palate abnormalities, sleep disturbances and feeding difficulties during infancy (Skjei et al., 2007; Ockeloen et al., 2015; Goldenberg et al., 2016; Low et al., 2016). Abnormal electroencephalogram (EEG) findings both with and without epileptic seizures are often found (Brancati et al., 2004; Skjei et al., 2007; Kim et al., 2015; Goldenberg et al., 2016; Low et al., 2016). Neuroimaging studies show small cerebelli (Tunovic et al., 2014; Kim et al., 2015; Goldenberg et al., 2016), enlargement of the ventricles, and/or white matter anomalies in half of the patients included in these imaging studies (Skjei et al., 2007; Miyatake et al., 2013; Low et al., 2016).

As to psychopathology, the limited amount of research on KBG syndrome mainly comprises case studies, presenting anecdotal evidence of both children and adults with ADHD symptoms as well as autism related problems (Brancati et al., 2004; Skjei et al., 2007; Hah et al., 2009; Ockeloen et al., 2015; Goldenberg et al., 2016; Low et al., 2016). Incidentally reported are anxiety, as well as aggressive and compulsive behavior (Maegawa et al., 2004; Lo-Castro et al., 2013; Ockeloen et al., 2015; Goldenberg et al., 2016; Low et al., 2016). Even less is known about the phenotypical presentation in terms of cognitive functioning. The establishment and understanding of neurocognitive deficits that may underlie or contribute to psychopathology, can provide directions for personalized patient care regarding education, daily living and treatment. In the above-mentioned studies delayed speech and motor development (Kim et al., 2015; Goldenberg et al., 2016; Low et al., 2016) as well as mild to moderate intellectual disabilities have often been mentioned (Sirmaci et al., 2011; Ockeloen et al., 2015; Goldenberg et al., 2016), however cases with borderline to normal intelligence levels have also been described (Oegema et al., 2010; Walz et al., 2015; Goldenberg et al., 2016). Although intellectual disabilities have nearly always been reported in case studies, these classifications were only sporadically substantiated by systematic measurements of intelligence and neurocognitive functions. Up till now, only two case studies that both include two patients, have used cognitive tests in patients with KBG syndrome (Hah et al., 2009; Lo-Castro et al., 2013), suggesting problems in executive functions and visuo-spatial abilities. However, no correction for intelligence levels or comparison with IQ-matched individuals was mentioned. Comparison of patients with ID to controls with an equal level of general intelligence is essential for adequate interpretation of neuropsychological data as patients with ID will likely deviate on all domains of cognitive functioning compared to healthy controls. Specific cognitive deficits related to KBG syndrome would otherwise remain unnoticed. Potential cognitive deficits can only be identified as a specific characteristic of KBG syndrome when these deficits are reflected by differences in cognitive performance between individuals with KBG syndrome and individuals with a similar general level of intellectual functioning.

Thus, identification of the general level of intellectual functioning (total intelligence coefficient, a composite score reflecting achievements on all 10 subtasks of the intelligence battery), is a prerequisite to the identification of a specific neurocognitive profile in patients with KBG syndrome. Subsequently, neuropsychological screening based on exploration of intelligence profiles may then identify more specific deficits in information processing. Exploration of the intelligence profiles includes the comparison of achievements on different subtests within the intelligence battery, which all reflect different aspects of cognitive functioning. Establishment of strengths and weaknesses in cognitive functioning may provide direction for education, employment and therapy in patients with KBG syndrome. As mentioned above, in order to explore these potential deficits in information processing related to KBG syndrome, a comparison with control patients with similar levels of intelligence is necessary. The aim of the current study is precisely this and strives to explore the intellectual profiles of KBG syndrome in a larger, substantial cohort of patients (*n* = 18), compared with a suitable control group, which includes a mixed group of patients with different genetic syndromes other than KBG syndrome who also have an ID/DD and display behavioral difficulties.

## Materials and Methods

### Participants

The subjects of this study were 18 patients with KBG syndrome, all of whom had a confirmed mutation in the *ANKRD11* gene, and 17 control patients with an established ID/DD and genetic diagnoses other than KBG syndrome, hereinafter referred to as “patient genetic control group” (PGC). Genetic aberrations in the PGC group included patients with de novo gene mutations (*SIN3A*, *k* = 2; *TCF20*, *k* = 2; *DLG3; RIP12*; *KDM5; KMT2*) and microduplications (*PMP22*; chromosome 17; parts of chromosome 19) as well as microdeletions (18p; 21q22.2q22.3). All were approached for the study by the Department of Human Genetics of the Radboud University Nijmegen Medical Centre, and participated voluntarily. Exclusion criteria for both groups were: an auditory or visual handicap, inability to speak, sit in a chair or hold a pencil. The study was approved by the Central Committee on Research Involving Human Subjects region Arnhem-Nijmegen (NL43187.091.13), and written informed consent was obtained from all participants, or their legal representatives.

Within the group of KBG patients, four patients had a history of epileptic seizures. Furthermore, although hearing difficulties were present as a result of regular ear infections (*k* = 2), partial hearing loss (~30%; *k* = 3) or solely loss in one ear (*k* = 2), sufficient hearing during the assessment was affirmed. Four patients are related (mother, her two daughters and her grandson). The molecular and phenotypical characteristics of six patients have previously been reported (Ockeloen et al., 2015).

In line with the norm groups of both Wechsler scales (for WISC-III 6–17 years of age and for WAIS-IV >16 years of age), both the KBG group and the PGC group were subdivided into two groups based on age, at 16 years and 6 months of age (KBG-children, KBG-adults, PGC-children, PGC-adults). For a description of demographical variables, see Table 1. No significant age differences were present between both groups of children (*t*_(22)_ = 1.24, *p* = 0.23, *d* = 0.05), and both groups of adults (*t*_(9)_ = 1.54, *p* = 0.16, *d* = 0.10).

**Table 1:** Demographical variables of PGC and KBG groups.

	*N (males)*	Age *(SD; range)*
KBG-c	12 *(6)*	9.1 *(2.4)*
KBG-a	6 *(1)*	34.8 *(17.4)*
PGC-c	12 *(8)*	10.6 *(3.4)*
PGC-a	5 *(4)*	22.4 *(4.8)*

*KBG-c, children with KBG syndrome; KBG-a, adults with KBG syndrome; PGC-c, patient genetic controls children; PGC-a, patient genetic controls adults*.

### Materials

In patients and controls below the age of 16 years and 6 months (KBG-c and PGC-c, respectively), intelligence was assessed with the Dutch Version of the Wechsler Intelligence Scale for Children Third Edition (WISC-III; Wechsler, 2005). In the adult groups (KBG-a and PGC-a), the Dutch Version of the Wechsler Adult Intelligence Scale Fourth Edition (WAIS-IV; Wechsler, 2012) was administered. Besides global intelligence, four specific subtests of the Wechsler scales were selected to screen cognitive functioning according to the four Wechsler subtest results, namely speed of information processing, working memory, verbal comprehension and perceptual reasoning. Speed of information processing was estimated by the subtest Coding, in which the patient has to pair symbols with numbers within a specified time limit. Furthermore, working memory was tapped by the subtest Arithmetic. Verbal comprehension and perceptual reasoning were measured by Vocabulary and Block Design respectively. To assess Vocabulary, a patient names the object presented visually (for picture items) and defines words that are presented visually and orally. In Block design the patient views a model and a picture, or a picture only and uses red-and-white blocks to recreate the spatial design.

### Statistical Analyses

For global intelligence, total intelligence coefficients were calculated based on the norm groups of the Wechsler scales. Additionally, for screening of cognitive functioning based on intelligence profiles, raw tests scores of the subtest Coding, Arithmetic, Vocabulary and Block Design were transformed into scaled Wechsler Standard Scores (WSS, range 1–19), based on the norm groups of the Wechsler scales conform the standard test procedure as described in the Wechsler test manuals. Subsequently, differences in TIQ and WSS between four groups (KBG-c, KBG-a, PGC-c, PGC-a) were examined by *t*-tests and General Linear Model (GLM) multivariate analysis of variance (MANOVA; Wilks’ Lambda) and standardized effect sizes respectively (partial eta squared) were computed. Additionally, contrasts tests were executed to identify the cognitive domains that contributed to any group differences.

## Results

In Table 2 mean total intelligence coefficients (TIQ) as well as mean WSS per subtest of both KBG and PGC groups are presented. Performances in terms of TIQ and WSS show dispersion between the individual patients in all four groups. Classifications of the total intelligence range from a moderate ID (TIQ: 45) to normal intelligence (TIQ: 99), see Table 3 for frequency distribution per group. No significant differences were found on TIQ between KBG-c and PGC-c (*t*_(22)_ = 0.23, *p* = 0.82, *d* = 0.41). Likewise, KBG-a and PGC-a did not differ in TIQ (*t*_(9)_ = −0.20, *p* = 0.85, *d* = 0.01). For the subtests, WSS range from “far below average” (WSS: 1) to “above average” (WSS: 13) with a relatively high amount of (very) low scores in children of both groups.

**Table 2:** Intelligence coefficients^1^ and Wechsler standardized scores^2^ per group.

	*Mean (SD)*			
	KBG-c (*n* = 12)	KBG-a (*n* = 6)	PGC-c (*n* = 12)	PGC-a (*n* = 5)
TIQ	66.5 (15.6)	62.5 (7.1)	68.0 (16.3)	63.6 (11.5)
Block design	3.9 (2.7)	5.2 (1.2)	5.4 (3.2)	5.6 (1.1)
Coding	6.0 (3.2)	2.8 (1.2)	4.1 (2.8)	2.8 (1.9)
Vocabulary	4.7 (3.8)	4.3 (1.4)	5.3 (3.8)	4.2 (2.2)
Arithmetic	4.3 (3.0)	4.0 (0.9)	3.5 (2.4)	3.8 (1.8)

*^1^M = 100, SD = 15; ^2^M = 10, SD = 3 TIQ, total intelligence coefficient; KBG-c, children with KBG syndrome; KBG-a, adults with KBG syndrome; PGC-c, patient genetic controls children; PGC-a, patient genetic controls adults*.

**Table 3:** Frequency distribution Intelligence classifications per group.

	*N*			
	KBG-c	KBG-a	PGC-c	PGC-a
Average	1	0	1	0
Low average	2	0	2	0
Borderline	1	1	1	2
Mild Intellectual Disability	6	5	6	3
Moderate Intellectual Disability	2	0	2	0

*KBG-c, children with KBG syndrome; KBG-a, adults with KBG syndrome; PGC-c, patient genetic controls children; PGC-a, patient genetic controls adults*.

GLM multivariate analyses of the WSS showed an overall significant difference between the four groups (*F*_(12,74)_ = 3.07, *p* = 0.001, *η*^2^ = 0.30). However, additional comparison of results on the individual subtests did not display significant specific group differences. So, group performances did not differ for the subtests Block Design (*F*_(3,31)_ = 0.90, *p* = 0.454, *η*^2^ = 0.08), Vocabulary (*F*_(3,31)_ = 0.17, *p* = 0.919, *η*^2^ = 0.02) and Arithmetic (*F*_(3,31)_ = 0.25, *p* = 0.864, *η*^2^ = 0.02). A marginal significant difference was found on Coding (*F*_(3,31)_ = 2.81, *p* = 0.056, *η*^2^ = 0.21). Planned contrasts revealed that this difference is possibly due to significantly higher performance of KBG-c when compared to KBG-a (*p* = 0.023).

## Discussion

Intellectual functioning in KBG syndrome was investigated in a case-control study, that included patient genetic controls. Total IQ of patients with KBG syndrome varied from moderate ID to normal intelligence. When compared with PGC, there were no significant differences on the four Wechsler subtest results. According to the results on Coding, there was a marginal significant difference in speed of information processing, which was mainly constituted by differences in functioning between KBG-c (faster) and KBG-a (slower).

The established level of intellectual functioning is in line with previously performed descriptive studies on KBG syndrome (Sirmaci et al., 2011; Ockeloen et al., 2015; Goldenberg et al., 2016). Intelligence in the current study ranges from moderate ID to normal intelligence, with most patients having a mild ID. Heterogeneity within both KBG-c and KBG-a groups was evident regarding both global intelligence and intelligence subtest profiles. In contrast to what may have been expected, Wechsler subtest results of both KBG groups were not significantly different from those of both control groups, meaning that patients with KBG syndrome did not display specific strengths or weaknesses in measures of speed of information processing, working memory, verbal comprehension or perceptual reasoning compared to the control patients with other genetic syndromes.

Although patients with KBG syndrome clearly deviated from the healthy population in both the general level of intelligence and intelligence profiles (as reflected in the low TIQ and WSS scores), there are no indications for more specific weaknesses in cognitive functioning in KBG syndrome. Previously, the behavioral phenotype of KBG syndrome has often been characterized as “ADHD-like behavior”. Given the fact that patients with ADHD have demonstrated impairments in speed of information processing and working memory in Wechsler profiles (Mayes and Calhoun, 2006; Theiling and Petermann, 2016), specific deficits in these domains could also be expected in patients with KBG syndrome. However, this study indicates that the behavioral phenotype of KBG syndrome is not directly related to specific impairments in speed of information processing or working memory performance. Alternatively, neurocognitive functions such as attention and executive functioning (EF) may underlie or contribute to this phenotype, regarding the fact that prominent neuropsychological theories on ADHD state that this psychopathology is related to specific deficits in EF. Meta analysis of case-control studies of patients with ADHD, for example, display significant weaknesses in several key EF domains like response inhibition, vigilance and planning (Willcutt et al., 2005). As Wechsler batteries primarily tap into crystallized intelligence and do not specifically measure attention and EF (van Aken et al., 2014, 2017), future studies on KBG syndrome could focus on more specific aspects of neurocognitive functioning.

In conclusion, the current study demonstrates a wide range in the level of intellectual functioning in patients with KBG syndrome, varying from a moderate ID to average levels of intelligence, with a majority of patients demonstrating a mild ID. Furthermore, when compared with PGC, patients display quite similar Wechsler subtest results, which indicates that specific behavioral problems associated with KBG syndrome are not related to a specific intelligence profile. In order to further clarify the KBG behavioral phenotype, future studies should include more specific measures of neurocognitive functioning including measures of attention and EF.

## Author Contributions

LCMD, JIME, TK and EW designed and planned the study. LCVD and AGB-R acquired the data and performed neuropsychological assessments. WO analyzed the neuropsychological data and contributed to the interpretation of the results. CWO and TK diagnosed and recruited the patients and contributed to the interpretation of the genetic analyses. LCMD, EW and JIME drafted the manuscript. TK, WO, AGB-R and CWO critically reviewed the manuscript. All authors read and authorized the final version of the manuscript.

## Conflict of Interest Statement

The authors declare that the research was conducted in the absence of any commercial or financial relationships that could be construed as a potential conflict of interest.
